# Metabolic Profiling of Bulgarian Potato Cultivars

**DOI:** 10.3390/foods11131981

**Published:** 2022-07-04

**Authors:** Nasya Tomlekova, Petko Mladenov, Ivayla Dincheva, Emilya Nacheva

**Affiliations:** 1 Maritsa Vegetable Crops Research Institute, Agricultural Academy, 32 Brezovsko Shossee Street, 4003 Plovdiv, Bulgaria; emnach@abv.bg; 2 Agrobioinstitute, Agricultural Academy, 8 Dragan Tzankov Boulevard, 1164 Sofia, Bulgaria; rubisko@abv.bg (P.M.); ivadincheva@yahoo.com (I.D.)

**Keywords:** antioxidants, GC-MS, flavonoids, metabolites, nutritive quality, phenolics, potato, *Solanum tuberosum* L.

## Abstract

Potatoes (*Solanum tuberosum* L.) are the fourth most economically important crop in the world. They have a short period of vegetation and are an excellent source of carbohydrates, amino acids, vitamins, organic acids, minerals and phenolics as antioxidant substances. Potato can be a major dietary source of various bioactive compounds. In this study, we applied gas chromatography coupled with mass spectrometry (GC-MS) metabolite profiling to classify eight Bulgarian potato cultivars bred in the Maritsa Vegetable Crops Research Institute (VCRI), Plovdiv, according to their metabolite contents. Altogether, we determine their flavonoids/phenolics to evaluate their nutritive quality for the breeding program with the target of determining strong health-promoting compounds. The “Kalina” cultivar is highlighted as the best one with the highest number of metabolites, containing 14 out of the 26 evaluated; it was selected as the highest-quality cultivar, compared with the other seven cultivars studied. According to the grouping of the cultivars in principal component analysis PCA, their positive distribution is explained mainly by them having the highest contents of aminobutyric and isocitric acids, methionine and alanine and lower levels of fumaric acid, pyroglutamic acid and glycine, in contrast to the cultivars distributed negatively, which had high contents of carbohydrates and relatively low contents of most of the amino acids. The highest number of amino acids was found in the cultivar “Kalina”, followed by “Perun” and “Bor”. The highest number of carbohydrates was found in “Pavelsko” and “Iverce”, while the prominent accumulation of organic acids was found in “Kalina”, “Bor” and “Rozhen”. The highest number of flavonoids in the flesh of the tubers was found in the cultivars “Nadezhda” and “Pavelsko”, followed by “Bor”. The highest ratio of flavonoids/phenolics in the flesh was found in “Pavelsko” and in “Nadezhda”, followed by “Iverce”.

## 1. Introduction

Potato (*Solanum tuberosum* L.) was known outside the Andes four centuries ago [1]; it is the fourth most important crop in the world after maize, wheat and rice [2] with the total world potato production 359 million tons in 2020. Of that, 112 million tons produced in the European Union [3]. It is a critical crop in terms of food security in the face of population growth and increased hunger rates [4]. Potatoes originate from South America, where the species diversity of the culture is concentrated [5]. Botanically they belong to the *Solanaceae*. There are over 180 species of wild relatives [5,6], primitive and cultivated potatoes [7], and the local forms and landraces [8], of the Andean region (Peru, Bolivia and Ecuador) exceed 4000 cultivars [9]. 

Today, potatoes are one of the most common crops, as they can be grown from sea level to 4700 m above sea level—from southern Chile to Greenland [6]. Potatoes as a raw material have significant impact on the food and biofuel industries. Their tubers contain an average of 25% dry matter, of which 12–15% is starch, 1–3% is protein and about 1% is mineral compounds [10,11]. Carbohydrates in potatoes are one of the main sources of muscle energy for humans, with a daily consumption of 200–300 g providing about 10 % of the physiological caloric needs of people engaged in physical labor [12]. Potatoes are rich in vitamins and minerals [13,14], and are one of the main sources of vitamin C, whose content in fresh tubers is about 11.4 mg per 100 g or 20% of the daily required levels of this vitamin [15]. The mineral compounds in fresh tubers, which include calcium, iron, iodine, sulfur, etc. [16], have the advantage of alkaline excess, which neutralizes the acid residue in the blood and protects the human body from atherosclerosis and premature aging [9]. Potatoes, similar to onion, show 94% antioxidant activity against hydroxyl radicals and almost completely neutralize the action of superoxide radicals. Their tubers are largely used as a source of different bioactive compounds, such as phenolic compounds, and especially flavonoids, synthetized by the potato plant as a protection response from bacteria, fungi, viruses, and insects [17]. These compounds play the role of antioxidants, but also that of bioactive compounds and exhibit health-promoting effects in humans. Phenolic acids are the most abundant phenolic compounds in potatoes [18]. Among them, chlorogenic acid, which is an ester of caffeic acid and quinic acid, constitutes 90% [19]. The major phenolics which have been quantified in potatoes are ferulic, gallic, and p-coumaric acid in potatoes, ranging from 0 to 5 mg/100 g dry weight [20], and the minor—syringic, vanillic, sinapic, and salicylic acid are – present in small quantities [21]. The most abundant flavonoids include flavanols and anthocyanins [22]. Flavonoids have high antioxidant activity against free oxygen radicals.

Recent technological advances in large-scale metabolic profiling have provided a modern technology platform widely applied in diagnostics and functional genomics and used for screening purposes. Metabolic profiling, in particular, the GC-MS approach, is widely used to examine high numbers of metabolites including amino acids (isoleucine, lysine and valine), organic acids, carbohydrates and sugar alcohols in potato tubers [23]. Establishing biomarkers through GC-MC contributes to the evaluation of economically important phenotypes. Genetically diverse potato cultivars show significant differences in the levels of many amino acids thus allowing their clustering [24]. Asparagine, fructose and glucose are important to appreciate tuber nutritive quality and have been examined during storage among different potato cultivars [25].

Being at the border area of optimal conditions for potato growing [26], Bulgarian cultivars have been developed with economically important characters, such as high productivity, earliness, resistance to nematodes and with good organoleptic properties [27]. The purpose of this study is to phenotype eight Bulgarian potato cultivars using the GC/MS approach for nutritive substances of potato tubers together with the evaluation of non-nutritive total phenols and flavonoids.

## 2. Materials and Methods

### 2.1. Plant Material

This study includes eight Bulgarian potato cultivars bred in VCRI, Plovdiv (“Iverce”, “Nadezhda”, “Orfei”, “Perun”, “Pavelsko”, “Rozhen”) and the Experimental Potato Station, Samokov (“Kalina”, “Bor”). The cultivars tested show a wide range of phenotypic variation [27,28,29,30,31], summarized in Table S1.

Plants of all the eight cultivars were grown in four replications (100 plants per replication) in the field of the VCRI under long-day conditions (16 h of light, 25 °C) in the first week of March.

### 2.2. Extraction, Derivatization of Polar Metabolites and Gas Chromatography Coupled with Mass Spectrometry Analysis

Freeze dried potato tubers (0.05 g) were grinded to fine powder with TissueLyser II (QIAGEN, Duesseldorf, Germany). A volume of 1.0 mL of methanol was added and the material was extracted for 30 min at 70 °C. Subsequently, 500 μL of chloroform was added and the material was extracted for further 5 min at room temperature with vortex. Then, 500 μL of distilled water was added and the extract is centrifuged at 12,000× *g*. The upper layer (polar fraction) was dried in CentriVap (Labconco, Kansas City, MS, USA), subsequently dissolved in 50 μL of pyridine (Sigma-Aldrich; St. Louis, MO, USA), and derivatized with 50 μL N,O-bis(trimethylsilyl)-trifluoroacetamide (BSTFA, Sigma-Aldrich) for 90 min at 40 °C. The GC-MS analysis was performed on a 7890A instrument coupled with MSD 5975C equipment (Agilent, Santa Clara, CA, USA) operating in Electron Impact (EI) mode at 70 eV. A HP-5 MS column (30 m× 0.25 mm× 0.25 μm) was used. The temperature program was: 100 °C for 2 min, 15 °C/min to 180 °C for 1 min, 5 °C/min to 300 °C for 10 min, run time 42.33 min. The flow rate of the carrier gas (helium) was 0.8 mL.min^−1^. A split ratio of 1:20 was used for the injection of 1 μL of the solutions. The compounds in the polar fraction were identified as trimethylsilyl (TMS) derivatives with the help of the NIST 08 database (NIST Mass Spectral Database, PC-Version 5.0—2005, National Institute of Standardization and Technology, Gaithersburg, MD, USA), and other plant-specific databases: the Golm Metabolome Database (http://csbdb.mpimp-golm.mpg.de/csbdb/gmd/home/gmd_sm.html accessed on 15 December 2021). In order to calculate the retention index RI (as Kovàts index) of each compound, a mixture of aliphatic hydrocarbons (C_8_-C_40_) (Sigma-Aldrich, St. Louis, MO, USA) is injected into the system under the above temperature program.

For the identification of amino acids, mixture of standard AA (Amino Acid Standard Solution Prod. No A 6407; Sigma-Aldrich, St. Louis, MO, USA) was used as well. Quantity of the identified metabolites were considered by percentage peak area which appeared at the total ion chromatogram (TIC) in GC-MS analysis.

### 2.3. Statistical Analyses

PCA was performed to summarize the variance in metabolic data. The imputed data matrix contained all potato cultivars and respective replicates (*n* = 4) as rows and metabolites as columns. Prior to PCA, the data matrices were log2-transformed and standardized to zero mean and unit variance. All data were analyzed with MATLAB software according to standard procedures. One-way ANOVA was performed for evaluation of significance of differences of metabolites between different potato cultivars. For visualization of the variance of each metabolite, box plot charts are used.

### 2.4. Extraction and Spectrophotometric Analysis of Phenolic Compounds

Determination of total phenolic concentrations in Bulgarian fruits and vegetables was conducted by using the Folin–Ciocalteu assay [32] according to the EN ISO/IEC 17025:2001 [33]. This is a UV–Vis spectrophotometry method, which is generally accepted in analytical practice and used as Bulgarian State Standard Method to determine the concentrations of total phenols. It was adapted and used for analysis of potato fruits. The total phenolic concentration of the samples is expressed as mg gallic acid equivalent/100 g fresh weight. All samples were assayed in triplicate. Total phenolic concentration of fruits and vegetables is expressed as mg gallic acid equivalents (GAE)/100 g fresh weight. They were calculated by a standard curve constructed by gallic acid.

### 2.5. Extraction and Spectrophotometric Analysis of Total Flavonoid Compounds

Determination of total flavonoid concentrations in Bulgarian fruits and vegetables was performed by using the aluminum chloride colorimetric assay [33] according to the Bulgarian State Standard Method EN ISO/IEC 17025:2001. It was adapted and used for analysis of potato fruits. Total flavonoid concentration of fruits and vegetables is expressed as mg catechin equivalent/100 g fresh weight. All samples are assayed in triplicate. They were calculated by a constructed standard curve of catechin solution.

## 3. Results

### 3.1. GC-MS Metabolite Profiling

**Figure 1:**
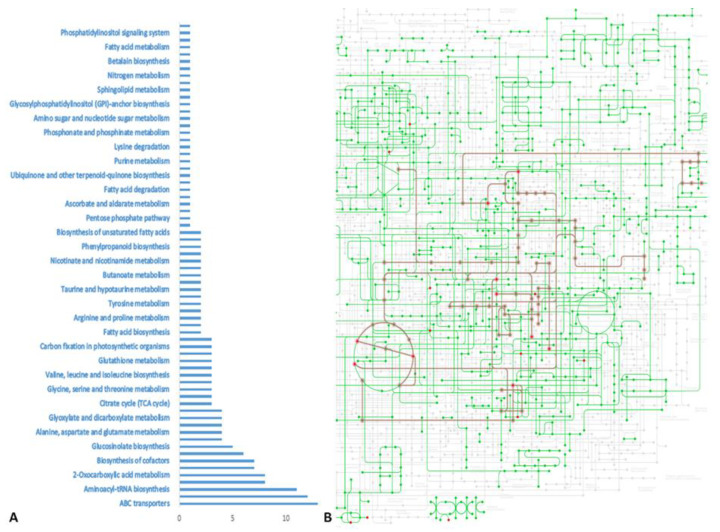
Pathway analysis. (**A**) KEGG list with enriched pathways. Bars for each pathway represented the number matched metabolites. (**B**) Visualization of KEGG metabolic pathways (green) and matched metabolites (red nodes). With red are highlighted the pathways with more than 3 matched queries.

From the conducted GC-MS metabolite profiling, we observed a total of 124 peaks in each chromatogram of each potato cultivar (Figures S1–S8). Furthermore, we identified and quantified 26 metabolites (Table S2) associated with different metabolic pathways of *Solanum tuberosum* in Kyoto Encyclopedia of Genes and Genomes (KEGG) with the provided online search tool. Sixty-eight pathways were enriched (Figure 1A), including ABC transporters, the biosynthesis of amino acids, aminoacyl-tRNA biosynthesis, D-amino acid metabolism, 2-oxocarboxylic acid metabolism, carbon metabolism, biosynthesis of cofactors, cyanoamino acid metabolism, glucosinolate biosynthesis, galactose metabolism, alanine, aspartate and glutamate metabolism, cysteine and methionine metabolism, glyoxylate and dicarboxylate metabolism, biosynthesis of various plant secondary metabolites, citrate cycle (TCA cycle), arginine biosynthesis, glycine, serine and threonine metabolism, monobactam biosynthesis, valine, leucine and isoleucine biosynthesis, phenylalanine metabolism and glutathione metabolism, with more than three associated queries (Figure 1B).

The list of amino acids quantified includes aspartic acid, isoleucine, leucine, methionine, proline, threonine, tyrosine, valine, glutamic acid, glycine, alanine, aspartic acid and phenylalanine, which are involved in pathways in potatoes such as amino acid metabolism, aminoacyl-tRNA biosynthesis, oxocarboxylic acid metabolism, phenylpropanoid biosynthesis, pantothenate and CoA biosynthesis, glucosinolate biosynthesis and the biosynthesis of cofactors. Amino acid derivatives such as pyroglutamic acid and non-proteinogenic amino acids such as α-aminobutyric acid were also found to be present in the Bulgarian cultivars. Very low quantities of urea were detected as well. Organic acids are represented by malic, fumaric and isocitric acids involved in carbon metabolism, glyoxylate and dicarboxylate metabolism, citrate cycle (TCA cycle) and pyruvate metabolism. Several isoforms of sucrose, glucose and fructose were detected and averaged means were used for each of these carbohydrates involved in starch and sucrose metabolism, fructose and mannose metabolism, galactose metabolism and the pentose phosphate pathway. Myo-inositol and two esters of fatty acids—stearic and palmitic—were detected in the polar extracts of the potato cultivars. Myo-inositol is involved in different pathways in potatoes, including ascorbate and aldarate metabolism, while stearic and palmitic acids are associated with fatty acid metabolism and cutin, suberine and wax biosynthesis.

**Figure 2:**
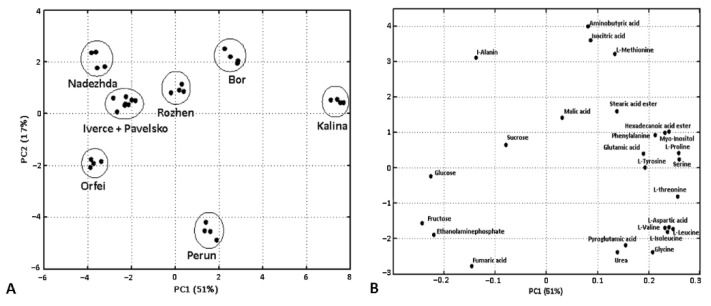
Principal component analysis of identified metabolites in different potato tubers. (**A**) PCA score plot. The replicates from each potato cultivar are represented with circlets. (**B**) PCA loading plot. The identified and quantified metabolites in each potato cultivar are represented with circlets.

The grouping of the cultivars in PCA (Figure 2A) is influenced by the variance in levels of amino acids and carbohydrates contributing in the the PC1 scale and the levels of organic acids and other metabolites in the PC2 scale (Figure 2B). The separation of cultivars in the positive scale of PC 1 is due to the high content of amino acids such as proline, serine, glutamate, phenylalanine and myo-inositol, in contrast to the lower content of carbohydrates. On the other hand, the cultivars grouped toward the negative scale of PC 1 show high content of carbohydrates and relatively low contents of most of the amino acids. The positive distribution of cultivars of PC 2 is explained mainly by them having highest contents of aminobutyric and isocitric acids, methionine and alanine, and lower levels of fumaric acid, pyroglutamic acid and glycine in contrast to these distributed negatively.

The position of “Bor” in the score plot is due to the higher percentages of TIC of isocitrate, aminobutyric acid, and methionine. It is also characterized by low levels of carbohydrates. The cultivars “Iverce” (fructose) and “Pavelsko” (glucose and sucrose) grouped toward the negative scale of PC1 show high carbohydrates and relatively low levels of most of the amino acids. An exception of the negative scale PC1 is thyrosine in “Iverce”. Surprisingly, the characteristic of the two “Iverce” and “Pavelsko” cultivars is a high percentage of TIC of carbohydrates and relatively low amino acids’ ones.

**Figure 3:**
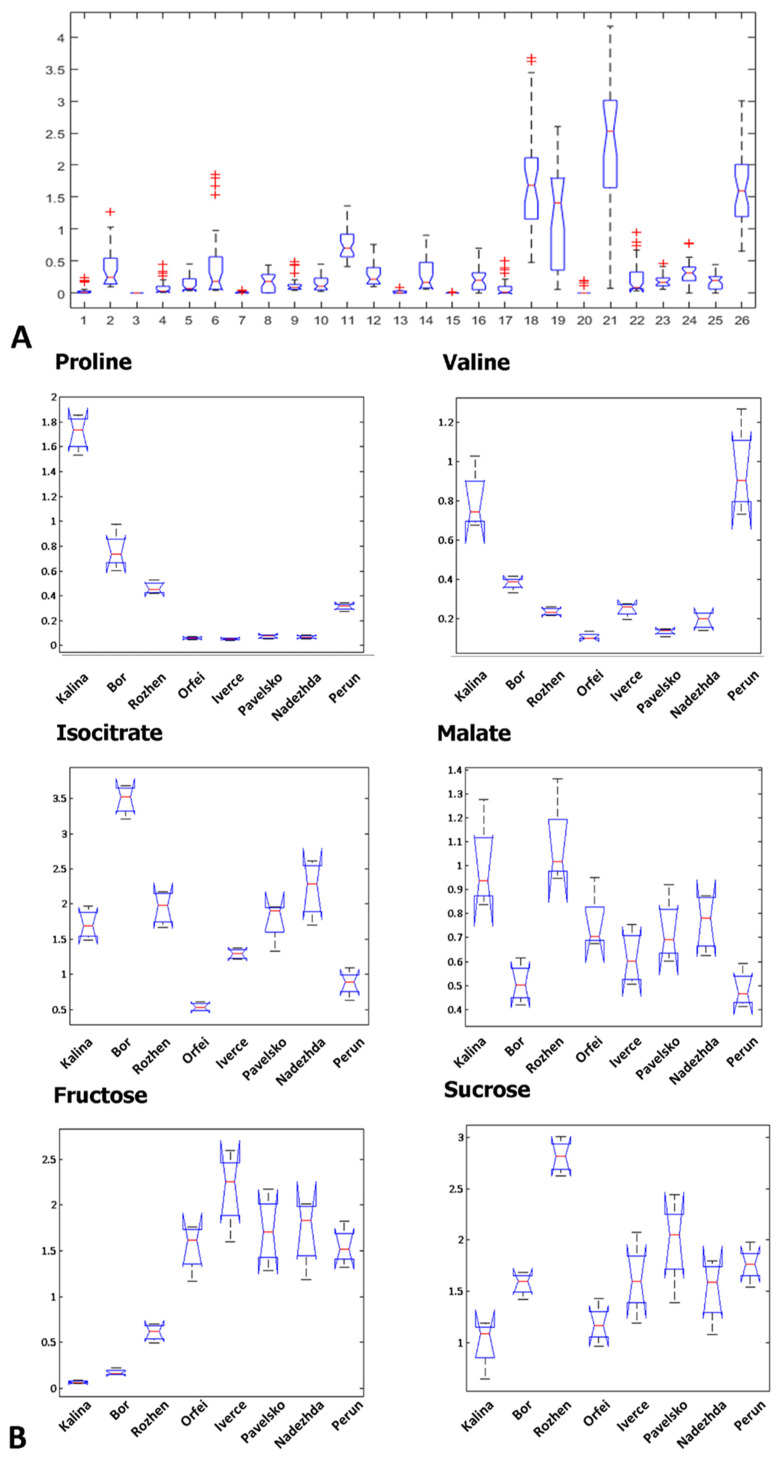
Variance in quantity of identified metabolites in different potato cultivars. Box plots represents the first and third quartile in blue, median mean with red and maximum and minimum means with bars. Outlayers are given with “+” in red (**A**) Box plot charts of means of each metabolite, measured in all potato cultivars. The number of metabolites in x axis are given as follows: (1) alanine; (2) valine; (3) urea; (4) leucine; (5) isoleucine; (6) proline; (7) glycine; (8) fumaric acid; (9) serine; (10) threonine; (11) malic acid; (12) asparatic acid; (13) methionine; (14) pyroglutamic acid; (15) 2-aminobutyric acid; (16) glutamic acid; (17) phenylalanine; (18) iso-citric acid; (19) fructose; (20) tyrosine; (21) glucose; (22) myo-inositol; (23) C16:0 (palmitic acid); (24) C18:0 (stearic acid); (25) ethanolamine phosphate;; (26) sucrose (**B**) Box plots of means of the metabolites with the higher variation between the potato cultivars. Each measurement is performed with four replicates.

“Rozhen” is characterized by the highest levels of sucrose, as well as the levels of malate and alanine. “Orfei” is separated from “Iverce”, “Pavelsko”, and “Nadezhda” according to the higher percent of TIC of glucose and fumaric acid quantified. The distribution of the data in Figure 3A of the most abundant metabolites shows high variability in proline, valine, fructose, sucrose, isocitrate, and malate. Proline and valine, in particular, are present in significantly higher percentages of TIC in “Kalina”, “Bor”, “Rozhen”, and “Perun” compared to the rest of the evaluated cultivars (Figure 3B). The established percentages of TIC of fructose in “Kalina”, “Bor”, and “Rozhen” are significantly lower than those observed in the other cultivars, whereas the highest levels of sucrose are observed in “Rozhen”. Out of all of the cultivars examined, “Bor” contains the highest isocitrate percent of TIC. Relatively high percentages of TIC of malate are detected in “Kalina” and “Rozhen”.

### 3.2. Spectrophotometric Analysis of Total Flavonoids and Phenolics Compounds

**Figure 4:**
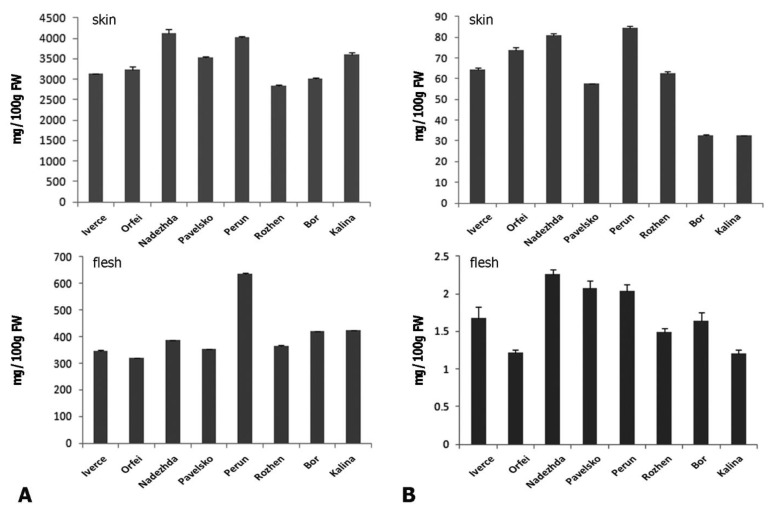
Concentration of total phenols and flavonoids in skin and flesh of potato cultivars. (**A**) Concentrations of total phenols. Upper panel represent total phenols measured in skin and lower panel phenols measured in flesh (**B**) Concentration of total flavonoids. Bars represent averaged means of three replicates and are given as mg per 100g of fresh weight of tubers Upper panel represent total flavonoids measured in skin and lower panel flavonoids measured in flesh.

The results of the study show that the eight Bulgarian potato cultivars have different levels of substances with biological activity—flavonoids and phenolics (Table 1, Figure 4).

The total phenols in the flesh of the studied tubers are between 318.12 mg/100 g FW in “Orfei” and 636.49 mg/100 g FW in potato tubers of the cultivar “Perun”. The tubers of the Bulgarian potato cultivars are characterized by a concentration of phenolics in the skin of 2846.88 mg/100 g FW of cultivar “Rozhen” to 4120.06 mg/100 g FW of cultivar “Nadezhda”. The concentrations of phenolics established in the skin of “Nadezhda” are close to those of the cultivar “Perun”. The highest flavonoid concentration of the tuber skin is quantified in “Perun”—84.37 mg/100 g FW—followed by “Nadezhda”—80.92 mg/100 g FW. The richest flavonoid concentration is in the flesh of “Nadezhda”—2.26 mg/100 g FW—followed by “Perun”—2.04 mg/100 g FW. The poorest is the flesh of the tubers of “Kalina”, with 1.21 mg/100 g FW, and “Orfei”—1.22 mg/100 g FW. The skin of “Bor” and “Kalina” has the lowest concentrations of flavonoids—32.69 and 32.54 mg/100 g FW. The best results regarding this ratio were found for the cultivars “Iverce”, “Orfei” and “Nadezhda” (Table 1). The highest ratio of flavonoids/phenolics in the flesh of “Pavelsko” is 0.006 and in “Nadezhda” is also 0.006, followed by “Iverce” with a 0.005 ratio in the flesh. The highest ratio of flavonoids/phenolics in the skin of “Rozhen” is 0.022, followed by “Iverce” (0.021) and “Perun” (0.021).

## 4. Discussion

In this study, we perform metabolic profiling of eight Bulgarian potato cultivars with a wide range of phenotypic variation previously established such as vegetative period, tuber shape, skin color, resistance to pathogens and resistance to tuber blight. Using GC-MS metabolite profiling we were able to measure the differences of 26 metabolites and classify these Bulgarian potato cultivars according to different organic acids, amino acids, and carbohydrates quantified in their tubers. Furthermore, we evaluated the total phenolic and flavonoid concentrations in the skin and flesh of tubers as the main antioxidant and bioactive compounds in potatoes.

Organic acids, amino acids, phenolic acids and reducing sugars determine the acidic reaction of potato tuber cell sap [34]. Here, we found variation in the accumulation of several compounds having an impact on the distribution of Bulgarian potato cultivars on the PCA scale (Figure 2). The organic acids assessed in potato tubers were between 0.4 and 1% of their fresh sap [34]. From the measured organic acids, we found highest variation in isocitrate and malate among the Bulgarian cultivars. “Bor” showed the highest concentration of isocitrate and low amount of malate, while “Perun” had a low level of both isocitrate and malate. “Kalina” and “Rozhen” showed the highest level of malate while “Orfei” showed the lowest level of isocitrate (Figure 3). It is known that levels of organic acids and especially citrate and isocitrate have an impact on the darkening of tuber flesh [34]; therefore “Bor” could tolerate prolonged storage periods without flesh darkening. The level of free amino acids in tubers is related to the technological quality of tubers and altogether with reducing sugars has to be considered for potato fried products [25]. It has been shown that the pretreatment of potatoes with exogenous proline inhibits browning of fresh-cut potatoes during cold storage [35]. We found the highest variation in proline and valine among potato cultivars. “Kalina”, “Bor”, “Rozhen” and “Perun” showed higher levels of proline and valine in contrast to the other cultivars (Figure 3). On the other hand, “Kalina”, “Bor” and “Rozhen” showed significantly lower levels of fructose compared to other cultivars. However, “Rozhen” showsed the highest level of sucrose (Figure 3). Fructose is an abundant reducing sugar in potatoes and can be accumulateed through the hydrolysis of sucrose [36]. Therefore, fructose has an impact on potato frying quality, considering that good frying quality is related with low levels of reducing sugars to avoid formation of dark pigments [37]. Two cultivars were distinguished by amino acids concentrations (“Kalina” and “Perun”). The “Kalina” cultivar was highlighted as the best by 14 out of the total 26 metabolites evaluated and “Perun” overcame the rest of the cultivars by 5 metabolites. It is selected from all cultivars as the highest-quality cultivar with the highest number of useful metabolites [18], and is incomparable with the other seven cultivars studied.

The levels of phenolic compounds in potatoes can vary greatly [38], depending on the color and the potato cultivars [39]. Phenols and flavonoids represent 200–500 mg and from 20 to 30 mg of tuber skin per 100 g fresh weight, respectively. The tuber flesh contains 10–60 mg/100 g fresh weight phenols and 0–3 mg/100 g fresh weight flavonoids [40,41]. “Nadezhda” surpasses the other potato cultivars in the concentrations of phenols and flavonoids, ranking second after “Kalina” and “Bor” in terms of phenolic concentration. The highest values of flavonoids in the flesh of the tubers were found in cultivar “Nadezhda” and “Pavelsko”, followed by “Bor” (Figure 4, Table 1). The flavonoids/phenolics ratio is an important indicator for the bioactive value of the potatoes. One of the best when the Bulgarian cultivars are compared is “Iverce” regarding the estimated ratios of flavonoids/phenolics in the flesh and in the skin, together. The ratios of “Nadezhda” is also very high in the flesh and in the skin and it overcame the other studied cultivars. The intake of phenolics above 20 mg daily dose is relatively toxic (Hussein Daood, personal communication, 2009).

We found that “Kalina”, “Bor” and “Rozhen” accumulate higher levels of amino acids such as proline and others, as well as of organic acids such as isocitric and malic acids, in contrast to their lower amounts of fructose, glucose and sucrose. This has the impact of of improving their prolonged storage suitability and good frying quality.

## 5. Conclusions

Our results show that the analyzed Bulgarian potato cultivars can be distinguished with GC/MS by the accumulation of different compounds such as organic acids, amino acids and sugars. The highest values of amino acids were observed in “Kalina”, followed by “Perun” and “Bor”. The highest carbohydrates were found in “Pavelsko” and “Iverce”, and the lowest ones were established in “Kalina” and “Bor”. “Bor” shows the highest concentration of isocitrate and a low amount of malate. “Kalina” and “Rozhen” show the highest levels of malate.

Furthermore, we also found differences in the accumulation of phenols and flavo-noids in potato cultivars. The highest values of flavonoids in the flesh of the tubers were found in cultivars “Nadezhda” and “Pavelsko”, followed by “Bor”. The highest ratio of flavonoids/phenolics in the flesh is found in “Pavelsko” and in “Nadezhda”, followed by “Iverce”.

## Figures and Tables

**Table 1:** Total phenolic and total flavonoid concentrations (mg/100 g FW) and their ratio in flesh and skin of fresh tubers in eight Bulgarian potato cultivars.

Potato Cultivar (Analysed Part of Tuber)	Phenolics (mg/100 g FW)	Flavonoids (mg/100 g FW)	Flavonoids/Phenolics Ratio
“Iverce” (skin)	3130.09 ± 11.9	64.61 ± 0.68	0.021
“Iverce” (flesh)	346.24 ± 1.7	1.68 ± 0.15	0.005
“Orfei” (skin)	3246.42 ± 70.68	74.03 ± 1.01	0.023
“Orfei” (flesh)	318.12 ± 0.47	1.22 ± 0.04	0.004
“Nadezhda” (skin)	4120.06 ± 10.48	80.93 ± 1.05	0.020
“Nadezhda” (flesh)	385.82 ± 1.83	2.26 ± 0.06	0.006
“Pavelsko” (skin)	3522.44 ± 27.07	57.51 ± 0.41	0.016
“Pavelsko” (flesh)	352.19 ± 2.04	2.07 ± 0.1	0.006
“Perun” (skin)	4015.26 ± 46.8	84.38 ± 0.86	0.021
“Perun” (flesh)	636.5 ± 1.52	2.04 ± 0.09	0.003
“Rozhen” (skin)	2846.89 ± 15.91	62.51 ± 1.09	0.022
“Rozhen” (flesh)	363.78 ± 3.65	1.49 ± 0.05	0.004
“Bor” (skin)	3004.95 ± 29.57	32.69 ± 0.39	0.011
“Bor” (flesh)	420.7 ± 0.97	1.64 ± 0.11	0.004
“Kalina” (skin)	3597.55 ± 57.56	32.54 ± 0.31	0.009
“Kalina” (flesh)	423.86 ± 1.54	1.21 ± 0.05	0.003

## Data Availability

The data presented in this study are available on request from the corresponding author.

## Supplementary Materials

The following supporting information can be downloaded at: https://www.mdpi.com/article/10.3390/foods11131981/s1. Table S1: Relative metabolites’ percent of TIC in tubers of potato cultivars. Figure S1. TIC of cultivar “Kalina”. Figure S2. TIC of cultivar “Bor”. Figure S3. TIC of cultivar “Rozhen”. Figure S4. TIC of cultivar “Orfei”. Figure S5. TIC of cultivar “Iverce”. Figure S6. TIC of cultivar “Pavelsko”. Figure S7. TIC of cultivar “Nadezhda”. Figure S8. TIC of cultivar “Perun”.

## Abbreviations

ANOVA	analysis of variance
AV	average value
DW	dry weight
GC	gas chromatography
FW	fresh weight
MATLAB	mathematical graphics programming
MS	mass chromatography
NIST	mass spectral database
PC	parent component
PCA	principal component analysis
PC1, PC2	principal component 1, 2
TMS	trimethylsilyl
TIC	total ion current
VCRI	Vegetable Crops Research Institute
