# Supplementary material for: Metabolic Profiling of Bulgarian Potato Cultivars
Source: Foods. 2022 Jul 4;11(13):1981. doi: 10.3390/foods11131981 (PMC9265564; doi:10.3390/foods11131981)
Supplement: Supplementary file 1 [file foods-11-01981-s001.zip › Supplementary_potatoes_[Tomlekova et al.]-1st revision.pdf]

Supplementary
Metabolic profiling of Bulgarian Potato Cultivars

Nasya Tomlekova [1], Petko Mladenov [2], Ivayla Dincheva [3] and Emilya Nacheva [4*]

Morphological and economical characteristics, metabolites of tubers of eight Bulgarian potato cultivars, and TIC of each studied potato cultivar

Table S1. Morphological and economical characteristics of tubers of Bulgarian potato cultivars.

Cultivar	Period of vegetation (days)	Consumer quality	Total tuber number/ plant	Average tuber weight (g)	Shape (index)	Shallownessof eyes (scale 0-9)	Total yield (kg/da)	References
"Iverce"	58.6	for fresh consumption, very good taste, free from discoloration after cooking	7.1	88.2	140.3	7.4	2339	Nacheva et al., 2010; Nacheva et al., 2013
"Nadezhda"	68.1	for fresh consumption, very good taste, free from discoloration after cooking	11.6	66.7	157.0	8.4	2933	Nacheva et al., 2010; Nacheva et al., 2013
"Orfei"	67.2	for fresh consumption, very good taste, non-discolorated flesh	10.8	70.5	110.1	8.2	2933	Nacheva et al., 2011; Nacheva et al., 2013
"Perun"	87.6	for fresh consumption and French Fries, very good taste, non-discolorated flesh	12.9	71.6	107.1	7.7	3438	Nacheva, 2004; Nacheva et al., 2013
"Kalina"	89.5	for fresh consumption and chips, free from discoloration after cooking	11.2	71.5	125.2	7.5	2853	Nacheva et al., 2010; Nacheva et al., 2013
"Pavelsko"	106.3	for fresh consumption and chips, free from discoloration after cooking	12.6	75.3	108.6	7.8	3224	Nacheva et al., 2014

"Rozhen"	98.7	for fresh consumption, French Fries and chips, free from discoloration after cooking	12.7	76.2	153.6	7.6	3509	Nacheva, 2004; Nacheva et al., 2013
"Bor"	113.7	for fresh consumption, very good taste, non-discolorated flesh	9.3	94.2	161.3	6.9	3254	Nacheva et al., 2010; Nacheva et al., 2013

Table S2. Relative metabolites' percent of TIC in tubers of potato cultivars.

#	RT	RI	matabolite	Kalina		Bor		Rojen		Orfey		Iverce		Pavelsko		Nadejda
			name	% of TIC	± s.e.	% of TIC	± s.e.	% of TIC	± s.e.	% of TIC	± s.e.	% of TIC	± s.e.	% of TIC	± s.e.	% of TIC
1	5,23	1097	Alanine 2TMS	n.d.	0,00	0,19	0,02	0,02	0,01	0,01	0,00	0,02	0,00	0,01	0,00	0,01
2	5,44	1208	Valine 2TMS	0,80	0,08	0,39	0,06	0,09	0,03	0,06	0,00	0,25	0,02	0,15	0,02	0,17
3	5,81	1238	Urea 2TMS	0,00	0,00	0,00	0,00	0,00	0,00	0,00	0,00	0,00	0,00	0,00	0,00	0,00
4	6,03	1265	Leucine 2TMS	0,12	0,01	0,06	0,00	0,02	0,00	0,02	0,00	0,03	0,00	0,01	0,00	0,02
5	6,22	1286	Isoleucine 2TMS	0,34	0,04	0,17	0,03	0,03	0,00	0,03	0,01	0,07	0,01	0,04	0,01	0,02
6	6,34	1293	Proline 2TMS	1,47	0,21	0,73	0,09	0,22	0,12	0,03	0,01	0,06	0,01	0,07	0,01	0,06
7	6,59	1299	Glycine 3TMS	0,01	0,00	0,01	0,00	0,01	0,01	0,00	0,00	0,00	0,00	0,00	0,00	0,00
8	6,79	1345	Fumaric acid 2TMS	0,00	0,00	0,01	0,00	0,17	0,03	0,19	0,06	0,20	0,04	0,00	0,00	0,29
9	6,84	1351	Serine 3TMS	0,42	0,04	0,21	0,04	0,06	0,01	0,05	0,02	0,05	0,01	0,05	0,01	0,06
10	7,11	1376	Threonine 3TMS	0,40	0,03	0,18	0,02	0,05	0,01	0,03	0,01	0,07	0,01	0,04	0,01	0,06
11	8,11	1477	Malic acid 3TMS	0,92	0,15	0,42	0,10	0,53	0,16	0,26	0,10	0,04	0,01	0,67	0,10	0,63
12	8,46	1509	Asparatic acid 3TMS	0,66	0,04	0,32	0,07	0,14	0,03	0,11	0,05	0,11	0,02	0,14	0,02	0,13
13	8,51	1513	Methionine 2TMS	0,09	0,00	0,03	0,00	0,01	0,00	0,00	0,00	0,01	0,00	0,00	0,00	n.d.
14	8,56	1517	Pyroglutamic acid 2TMS	0,44	0,05	0,11	0,03	0,22	0,11	0,03	0,01	0,02	0,00	0,07	0,02	0,09
15	8,64	1524	2-Aminobutyric acid 3TMS	0,01	0,00	0,00	0,00	0,00	0,00	0,00	0,00	0,00	0,00	0,00	0,00	0,00
16	9,66	1609	Glutamic acid 3TMS	0,66	0,02	0,37	0,08	0,11	0,06	0,04	0,00	0,17	0,04	n.d.	0,00	0,16
17	9,81	1623	Phenylalanine 2TMS	0,37	0,05	0,22	0,06	0,01	0,01	0,05	0,00	0,22	0,05	n.d.	0,00	0,01
18	12,50	1804	iso-Citric acid 4TMS	1,46	0,33	3,21	0,38	0,79	0,29	0,58	0,16	1,25	0,22	1,94	0,26	2,48
19	12,84	1856	Fructose 5TMS	0,07	0,01	0,22	0,08	0,57	0,05	1,03	0,13	2,17	0,21	1,72	0,19	1,71
20	14,38	1931	Tyrosine 3TMS	0,16	0,02	n.d.	0,00	0,02	0,00	0,17	0,02	0,54	0,11	n.d.	0,00	n.d.
21	14,90	1969	Glucose 5TMS	0,09	0,01	2,35	0,30	1,07	0,20	1,78	0,47	3,19	0,37	3,47	0,26	3,15
22	15,12	1992	myo-Inositol 6TMS	0,81	0,05	0,59	0,14	0,06	0,02	0,03	0,01	0,03	0,00	0,07	0,01	0,07
23	16,20	2042	C16:0 TMS (Palmitic acid)	0,38	0,06	0,30	0,06	0,07	0,01	0,05	0,01	0,11	0,02	0,17	0,02	0,12
24	19,49	2240	C18:0 TMS (Stearic acid)	0,69	0,08	0,41	0,06	0,11	0,04	0,05	0,00	0,21	0,03	0,32	0,02	0,24
25	20,40	2331	Ethanolaminephosphate	n.d.	0,00	n.d.	0,00	0,16	0,02	0,25	0,13	0,26	0,03	0,18	0,05	0,22
26	24,80	2620	Sucrose 8TMS	1,01	0,12	1,58	0,06	1,06	0,08	0,77	0,10	1,62	0,18	1,98	0,22	1,68

RT-retention time; RI-relative index; TIC-total ion chromatogramme; s.e.-standard error

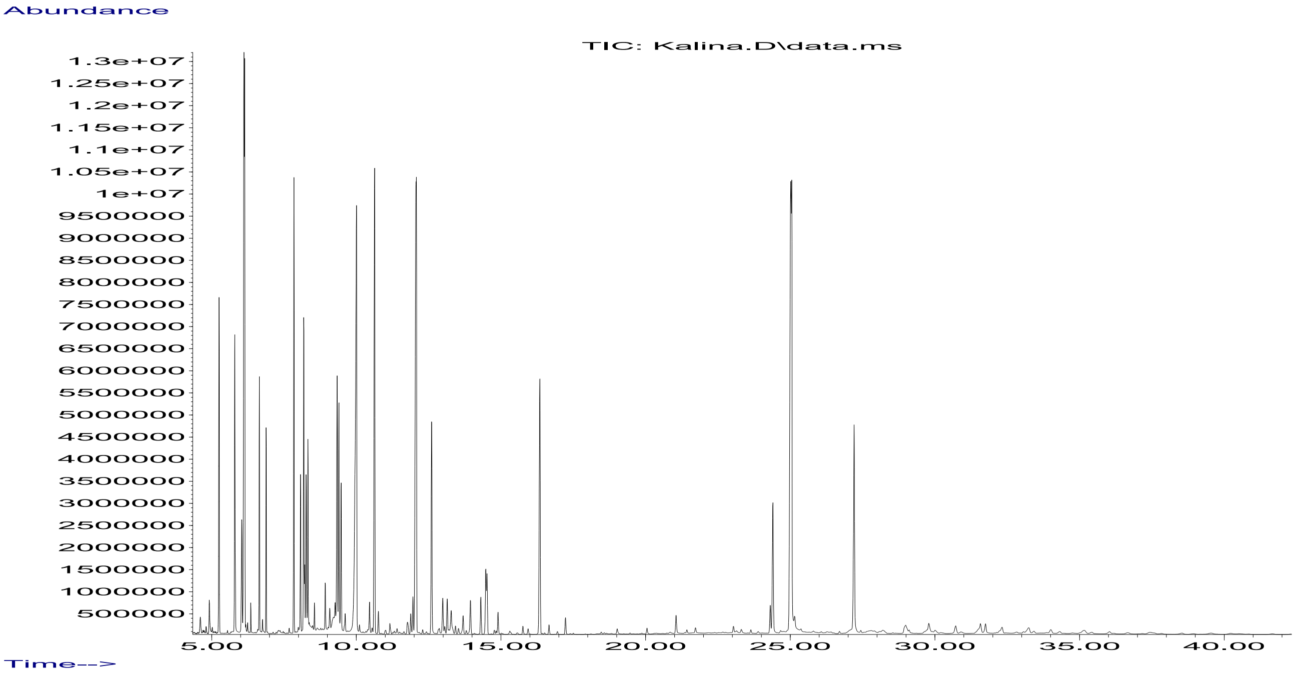

Figure S1. Percent of TIC of cultivar "Kalina".

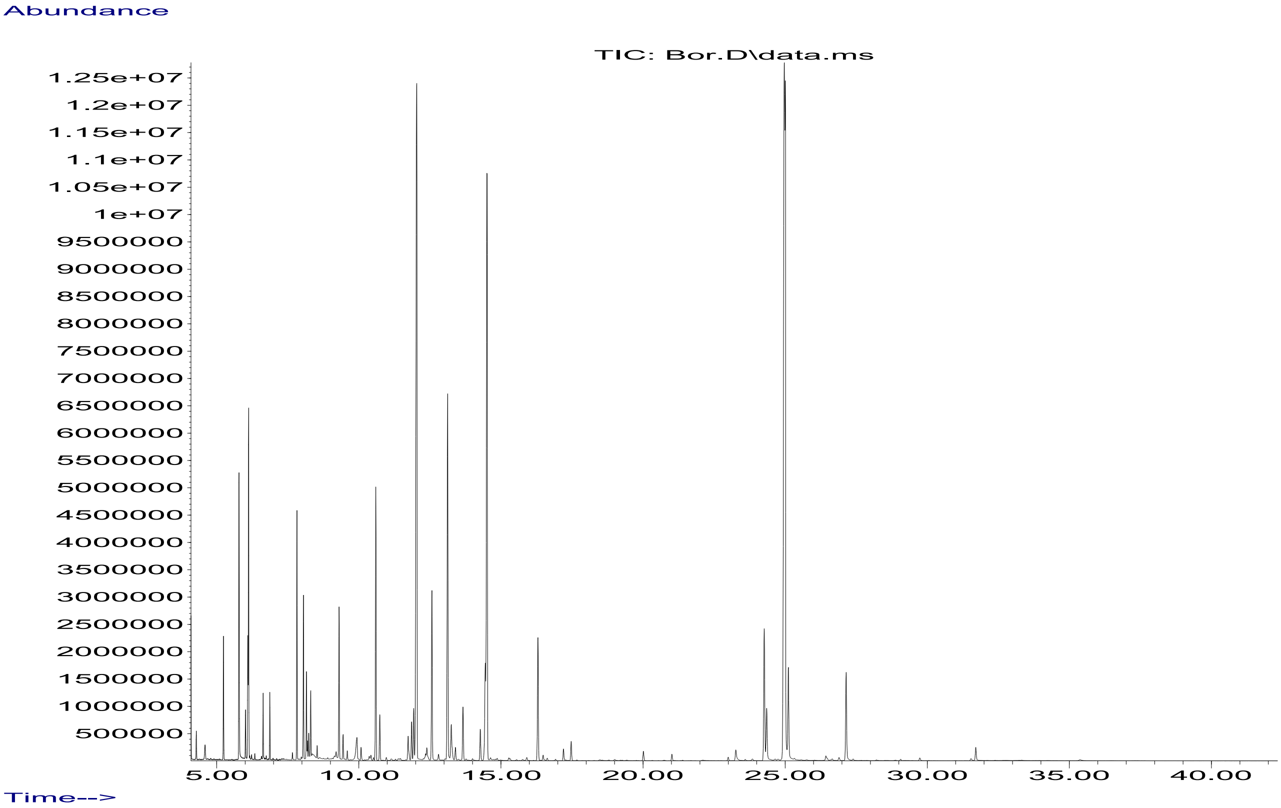

Figure S2. Percent of TIC of cultivar "Bor".

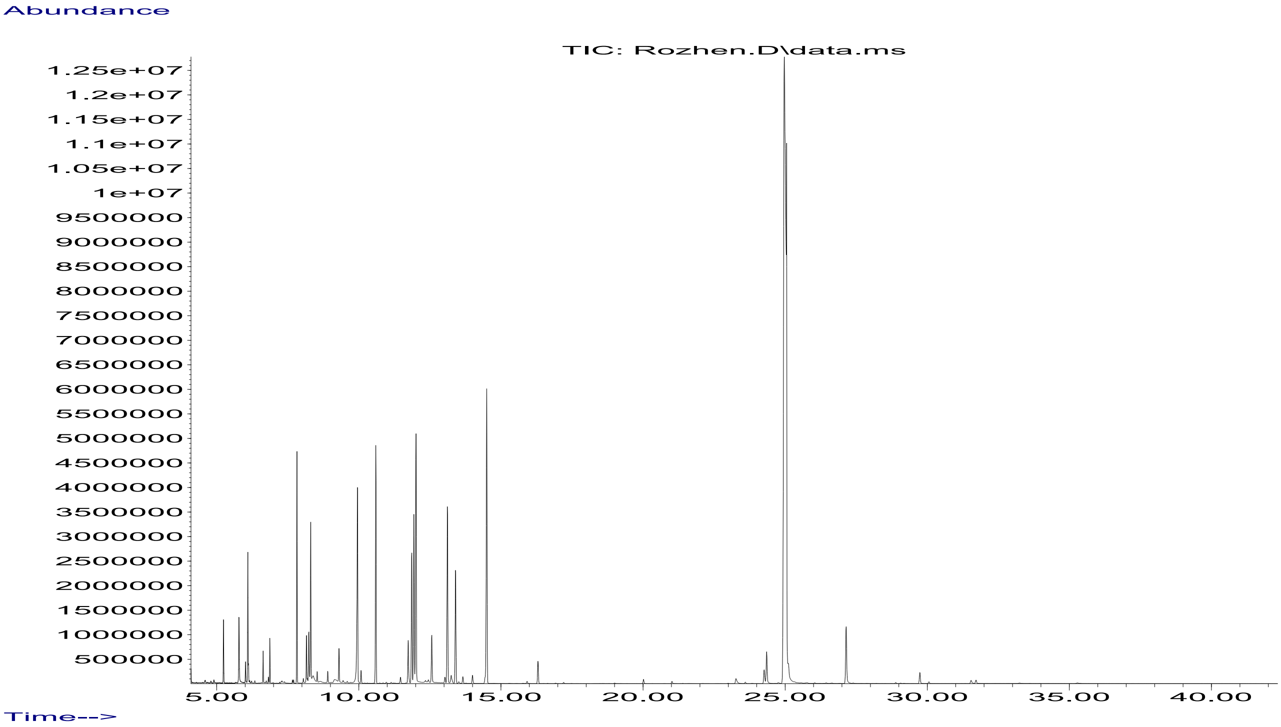

Figure S3. Percent of TIC of cultivar "Rozhen".

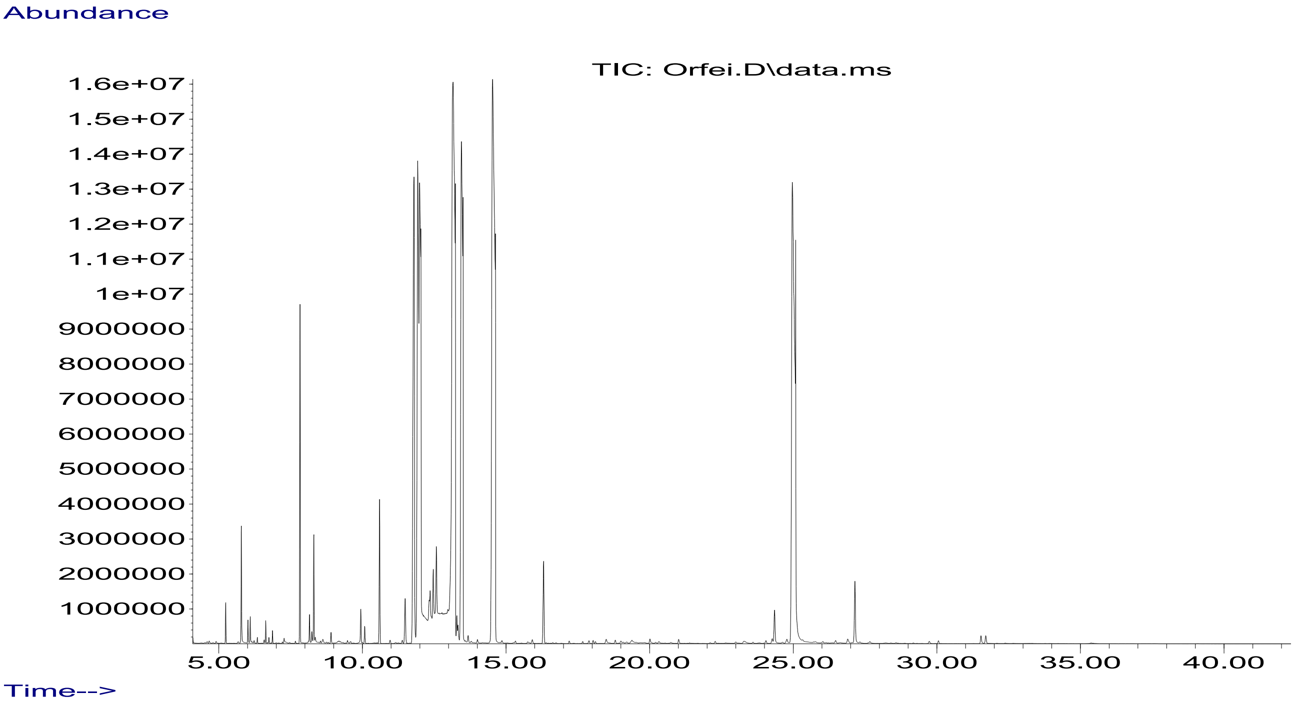

Figure S4. Percent of TIC of cultivar "Orfei".

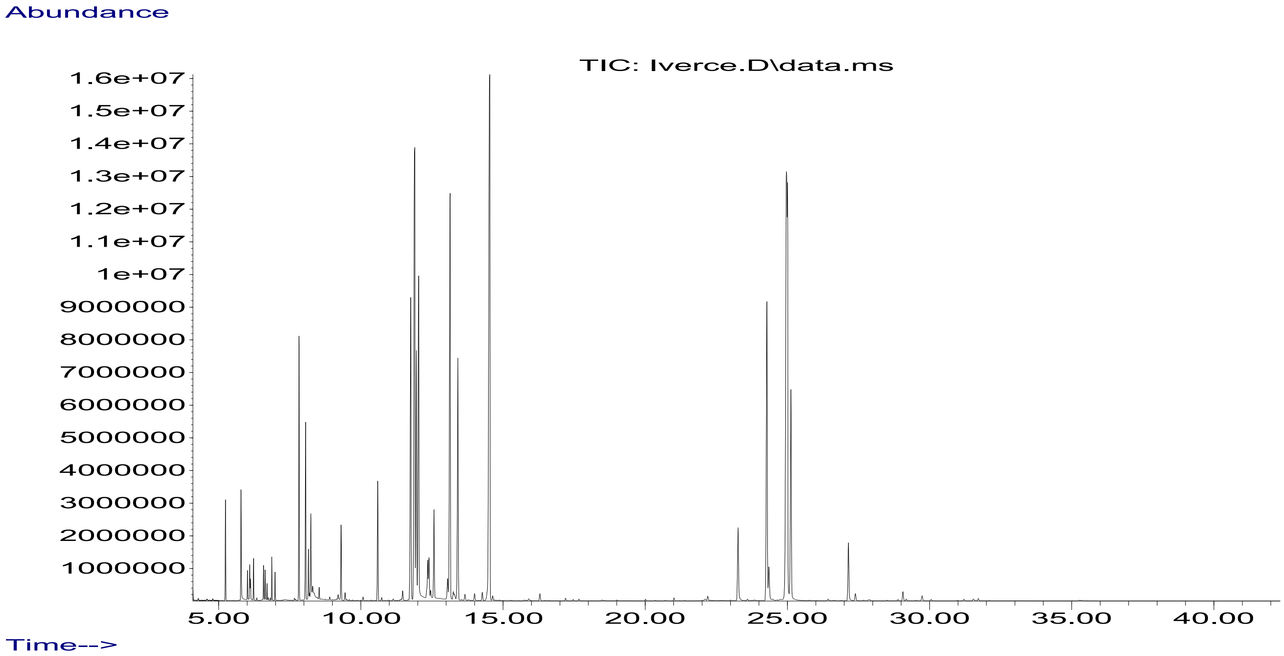

Figure S5. Percent of TIC of cultivar "Iverce".

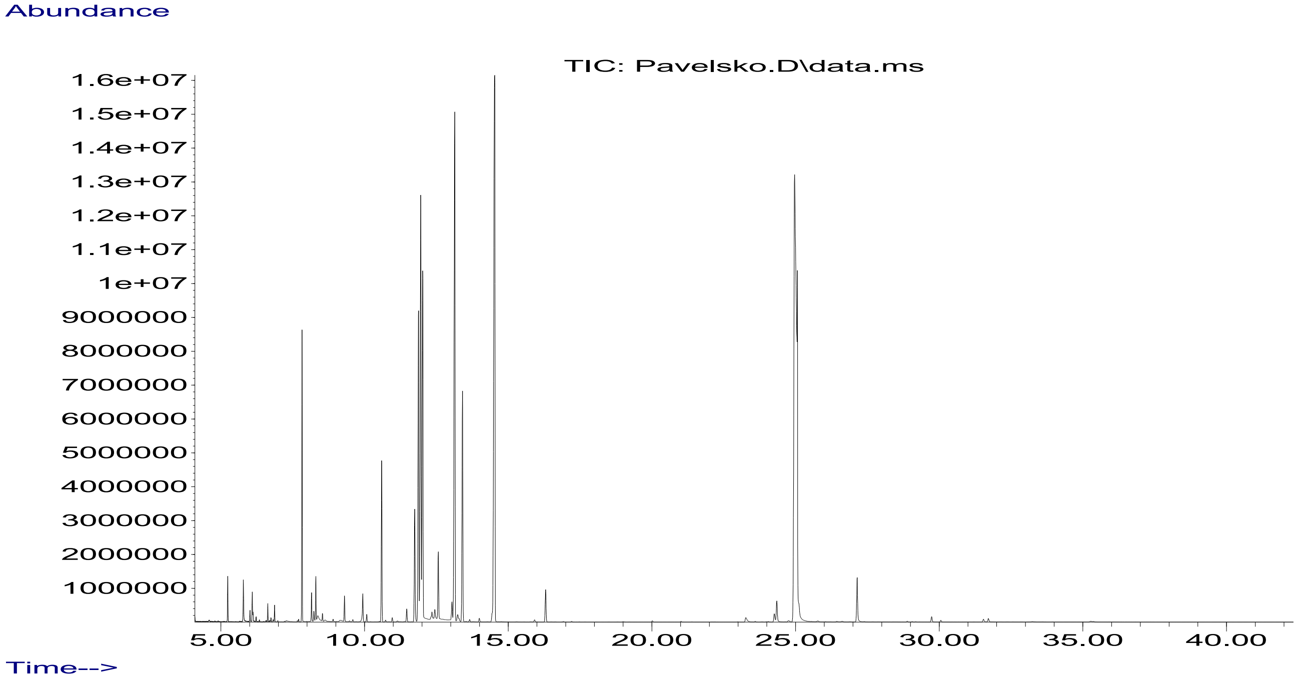

Figure S6. Percent of TIC of cultivar "Pavelsko".

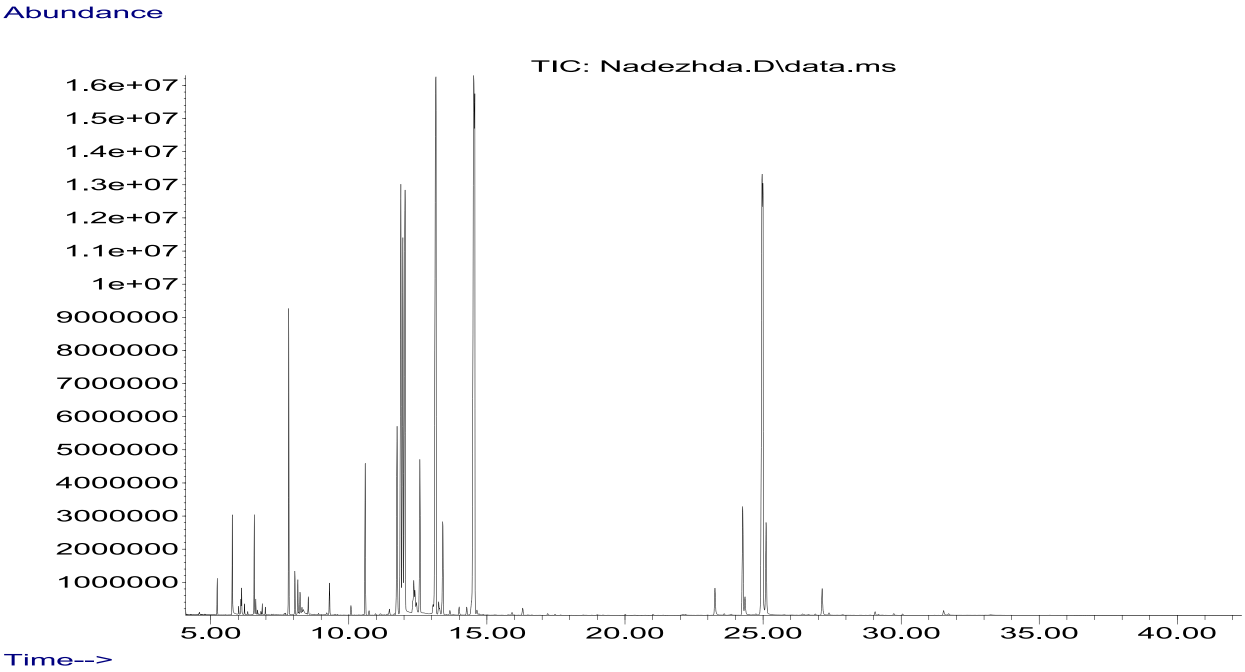

Figure S7. Percent of TIC of cultivar "Nadezhda".

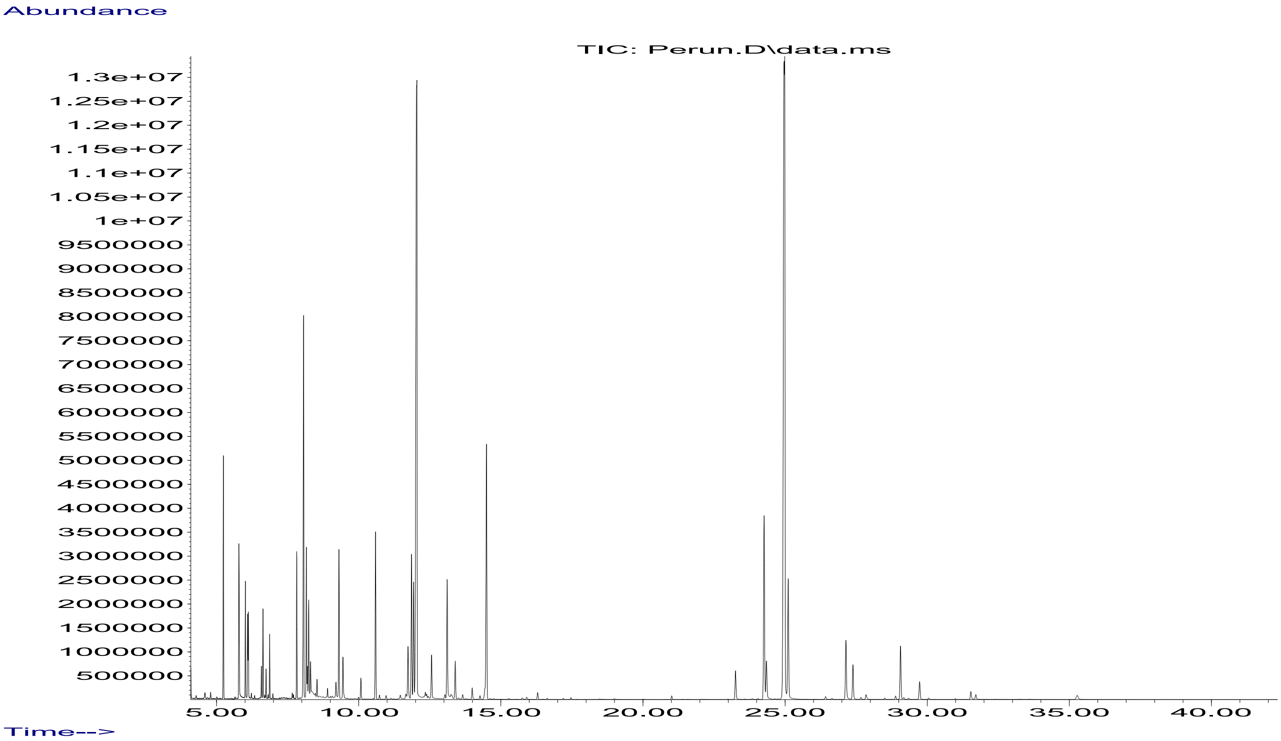

Figure S8. Percent of TIC of cultivar "Perun".
